# Modulation of the gut microbiota by prebiotic fibres and bacteriocins

**DOI:** 10.1080/16512235.2017.1348886

**Published:** 2017-01-01

**Authors:** Özgün C. O. Umu, Knut Rudi, Dzung B. Diep

**Affiliations:** ^a^ Faculty of Chemistry, Biotechnology and Food Science, Norwegian University of Life Sciences (NMBU), Ås, Norway

**Keywords:** Gut microbiota, prebiotics, bacteriocins, lactic acid bacteria

## Abstract

The gut microbiota is considered an organ that co-develops with the host throughout its life. The composition and metabolic activities of the gut microbiota are subject to a complex interplay between the host genetics and environmental factors, such as lifestyle, diet, stress and antimicrobials. It is evident that certain prebiotics, and antimicrobials produced by lactic acid bacteria (LAB), can shape the composition of the gut microbiota and its metabolic activities to promote host health and/or prevent diseases. In this review, we aim to give an overview of the impact of prebiotic fibres, and bacteriocins from LAB, on the gut microbiota and its activities, which affect the physiology and health of the host. These represent two different mechanisms in modulating the gut microbiota, the first involving exploitative competition by which the growth of beneficial bacteria is promoted and the latter involving interference competition by which the growth of pathogens and other unwanted bacteria is prevented. For interference competition in the gut, bacteriocins offer special advantages over traditional antibiotics, in that they can be designed to act towards specific unwanted bacteria and other pathogens, without any remarkable collateral effects on beneficial microbes sharing the same niche.

## Introduction

The development of the gut microbiota in mammals is an evolutionary progression that entails the gathering of microbes necessary for survival. The symbiosis between the gut microbes and the host is largely driven by complementary actions to extract nutrition and energy from foods [1]. The host provides indigestible foods to the gut microbes which, in return, ferment the food and provide the host with energy, e.g. in the form of short-chain fatty acids (SCFAs). In addition, the gut microbiota contributes to homeostasis and keeping pathogens away, the latter often through a synergy between the microbial activities of the gut microbiota and the immune system of the host [2].

Modulation of the gut microbiota has become a promising and important approach to improve host health as it protects the host from infections and diseases and produces important vitamins and SCFAs. The latter can serve as a useful energy source for host as well as playing a role in diverse physiological regulatory networks, including the immune system [3]. Diet, prebiotics, probiotics, antimicrobial agents and faecal transplantation are current strategies with great potential to modify and manipulate the gut microbiota. It has been shown that dietary interventions and long-term dietary habits can shape the gut microbiota in mice and humans [4,5]. The gut microbiota is a large consortium of many different organisms, including bacteria, archaea and yeasts. This consortium is highly structured as well as being dynamic, with small changes in diversity and composition along the timeline from birth to old age. When the normal gut microbiota is disrupted (dysbiosis) during disease conditions or during therapeutic treatments, it can worsen the host condition, for example, by allowing a bloom of unwanted bacteria or pathogens in the gut [6]. The cause-and-effect relationships between the microbiota and disease and disorders are not well known, such that it is still uncertain whether the changes in gut microbiota are a cause or an effect of the disease or disorder. However, there is a number of interventional studies aiming to develop strategies to modify the gut microbiota and reinstate the normal growth and activity of beneficial phylotypes [7–10].

Competition and cooperation between microbes in the gut are the major shaping forces of the communities in such a complex environment. The competition in the gut generally consists of two main types: exploitative competition, which entails limiting resources such as food components for others, and interference competition, which involves directly harming other strains via antimicrobial production [11]. In this review, we will discuss the properties of the gut microbiota and its manipulation to improve host health, with special focus on the use of some prebiotics to provoke exploitative competition, and bacteriocins to induce interference competition in the gut.

## The gut microbiota

### General characteristics

Defining the healthy microbiota forms a baseline from which to understand the microbiota–host interactions, as well as the associations with disease and disorders. It has been suggested that the human gut microbiota is normally dominated by three main bacterial clusters (i.e. enterotypes): *Bacteroides* (enterotype 1), *Prevotella* (enterotype 2) and *Ruminococcus* (enterotype 3). These enterotypes are driven by species composition and are not affected by gender, age or nationality [12]. However, this concept is much debated as the enterotypes are more gradient entities than discrete groupings among individuals [13]. Therefore, it is not possible to clearly define and classify the gut microbiota of each individual based on the enterotypes. Moreover, larger projects such as the US Human Microbiome Project [14] and the European Metagenomics of the Human Intestinal Tract [15], and many others [16], aim to identify healthy (normal) microbiota and have made considerable progress. However, in spite of the relatively well-organized structure of the main bacterial groups, it is difficult to define the composition of the normal or healthy microbiota owing to the complexity of the microbiota and its variation between and within individuals [17].

Interindividual variation is a commonly observed phenomenon in gut microbiota studies [17]. Although the causes of interindividual variations are not well known, factors such as diet, environment, host genetics and early microbial exposure are major determinants [14]. The variation in these factors for each individual may give rise to functional redundancy in gut microbiota, which results in diversity among individuals in the gut inhabitants, having the same role of keeping the normal gut functioning in each individual. Greater variations are usually encountered at deeper taxonomic levels rather than at phylum level [18].

In addition to interindividual variations, the diversity and composition of the gut microbiota vary within individuals throughout life. In the early stages of life, the microbiota has low diversity and low complexity. The initial colonizers in neonates, which include facultative anaerobes such as *Staphylococcus*, *Streptococcus, Enterococcus* and *Enterobacter* spp., create an environment favourable for subsequent obligate anaerobes, such as *Bifidobacterium*, *Bacteroides, Clostridium* and *Eubacterium* spp. [19,20]. Subsequently, the microbiota slowly develops into an adult-like, more diverse and stable state at around 3 years of age [21,22]. In the adulthood of healthy humans, the gut ecosystem is in a homeostatic equilibrium with temporal balance between different microbial groups, epithelial tissue of the intestine and the immune system of the host [21,23]. However, after approximately 65 years of age, the composition of the gut microbiota alters, with high interindividual variability, which is probably due to the physiological changes in the intestines that affect food digestion and absorption, and immune function [24]. Another possible reason for this alteration in gut microbiota is the frailty or health status of the host during ageing; however, it is still not clear whether the changes in microbiota are correlative with or causative for the poor health status during ageing [25].

### Response to environmental factors

Environmental factors such as host genetics, ageing, health, lifestyle, early colonization, use of antibiotics and diet can affect the diversity and composition of the gut microbiota [18,26]. However, the gut microbiota generally has a remarkable innate ability to resist such exposures and disturbances, a property known as resilience; therefore, the microbial community is usually drawn back to its original state before the disturbance [17,27]. Resilience is presumably a mechanism to suppress the blooms of subpopulations and/or to promote the growth of the desired bacteria [28]. An interactive network plays a role in this, where different groups of bacteria rely on each other and on signals from the host to survive and to persist within the host. However, the resilience of the communities varies and the recovery or disruption of the stable state may depend on the composition of the community, the type of disturbance and the length of exposure [17]. For example, long-term dietary interventions have mostly been shown to associate strongly with an altered pattern of certain enterotypes in the gut that overcomes resilience, while short-term dietary interventions normally do not change the microbiota composition [26,29]. However, it is still an open question and more studies are needed to determine whether the effects of long-term dietary intervention are reversible with readministration of the previous diet.

In addition to the resilience of the gut microbiota, the gut microbiome exhibits functional redundancy, which guarantees that the key functions are maintained for normal gut functioning [28,30]. These key functions are conserved among individuals and this set of genes is normally referred to as a core microbiome [1,31]. Various gut bacteria overlap functionally and they ensure that crucial functions are present in the gut (e.g. the bacterial housekeeping functions involved in metabolic pathways and the putative gut-specific functions involved in adhesion to host proteins), which contributes to robustness in the gut ecosystem [32].

## Important dietary components for the gut microbiota

### Dietary fibres

Diet is a factor for exploitative competition among the gut microbiota, providing nutrition for selective groups of bacteria, since it may act as a direct substrate for the microbiota via its indigestible ingredients and some by-products of digestion. Among the dietary components, dietary fibres are important as they cannot be digested or absorbed in the upper part of the gastrointestinal tract; however, they can be fermented by the gut microbiota in the lower part of the gastrointestinal tract (the large intestine).

Dietary fibres can be classified differently depending on their role in the plant, fibre components, polysaccharide type, simulated gastrointestinal solubility, site of digestion, digestion products and physiological properties [33]. Their beneficial returns mostly depend on their physicochemical characteristics, such as viscosity, solubility and fermentability [34]. Dietary fibres with different physicochemical properties have been studied for their effects on the feeding motivations of pigs [35]. Findings have shown that the viscous fibre pectin is the least satiating fibre, while the feeding motivation of the pigs is reduced by a bulky fibre, lignocellulose, and reduced more by a fermentable fibre, resistant starch [35]. Further studies have shown that increased levels of fermentable dietary fibre enhance satiety despite the lower metabolizable energy intake, and that the high-satiety effect of resistant starch can be attributed to its slow fermentation rate and high production of SCFAs, particularly butyrate [36].

### Prebiotics

Prebiotics are a subgroup of dietary fibres with resistance to gastric acidity and the digestive enzymes of mammals, and which confer a variety of health benefits [37,38]. The main characteristic of prebiotics is their selective stimulation of the growth and/or activity of intestinal bacteria associated with health and well-being [38]. The most well-known prebiotics are inulin, fructooligosaccharides (FOS), lactulose and galactooligosaccharides (GOS) [39]. Prebiotics have mostly been assessed for the enhancement of strains of *Bifidobacterium* and *Lactobacillus*, which produce lactate and acetate and contribute to the health of the host via fermenting prebiotics [40]. However, our increasing understanding of the gut microbiota indicates that the effect of prebiotics can be broader on the gut community, where competition and cooperation between bacteria are significant. Cross-feeding is a phenomenon where different microorganisms cooperate to efficiently utilize complex carbohydrates. For example, bifidobacteria are involved in cross-feeding with butyrate-producing bacteria for either utilization of partial breakdown products from dietary carbohydrates or consumption of the endproducts of fermentation, i.e. lactate and acetate [41]. These offer an insight into a broader prebiotic concept, where the aim is to monitor the beneficial changes in the gut microbiota as a whole community instead of focusing only on the intended target bacteria. In this concept, dietary carbohydrates that are fermented by the gut microbiota and enhance the production of beneficial metabolites in the gut are good candidates for being prebiotic [42]. These complex carbohydrates, which include resistant starch and plant cell-wall polysaccharides, constitute an important portion of the human diet, with a daily amount of 20–60 g reaching the colon, and act as fermentation substrates for the gut microbiota [43]. As a result of fermentation, SCFAs, mainly butyrate, acetate and propionate, are produced in the gut. They confer a number of health benefits on the host, such as acting as an energy source for colorectal tissues, stimulating cellular mechanisms that retain tissue integrity, contributing to the immune system and possibly having anti-inflammatory effects [3,13,44]. Furthermore, other organic acids such as formate, lactate and succinate, which are produced via fermentation of dietary fibres, lower the pH in the intestines and prevent the growth of pathogenic bacteria [45]. Therefore, selective predominance of the bacteria that produce these metabolites is valuable for the prebiotic traits of the complex carbohydrates.

### Complex carbohydrate-fermenting bacteria in human gut

Resistant starch and plant cell-wall polysaccharides, including cellulose, hemicelluloses (xylan, mannan, xyloglucan, β-glucan) and pectin, are non-digestible complex carbohydrates that influence microbial populations in the gut [46]. A variety of bacterial groups that carry genes encoding carbohydrate-active enzymes (CAZymes) in their genomes have been found in human gut, which suggests that these bacteria have the ability to degrade such complex carbohydrates [43,47]. These bacterial groups are mainly constituted of *Bacteroides* spp., which have the ability to degrade a broad repertoire of carbohydrates (e.g. cellulose, pectin, galactomannan, arabinogalactan, alginate and xylans) and a variety of *Firmicutes* spp. that ferment the complex carbohydrates to produce butyrate, or convert lactate to butyrate and propionate. The *Firmicutes* phylotypes include members of the Lachnospiraceae family, and species affiliated to the *Ruminococcus*, *Clostridium, Eubacterium* and *Lactobacillus* genera. Moreover, *Bifidobacterium* spp. that are affiliated to Actinobacteria also comprise species with genes encoding carbohydrate-active enzymes [43].

### Prebiotic carbohydrates

Resistant starch fulfils the definition of a prebiotic [37,48]. It provides prebiotic-type fermentation in the colon and confers many metabolic benefits, such as increasing bile salt turnover and laxation, reducing the risk of gastrointestinal tract cancers, and lowering the postprandial glucose response and blood lipid levels [48,49]. Moreover, it contributes to epithelial cell growth and proliferation by increasing the butyrate concentration via its fermentation by the gut microbiota [50]. There are different types of resistant starch, which have been defined based on their physicochemical properties [51]. The metabolic benefits and the group of bacteria in the gut that respond to resistant starch vary depending on the type of resistant starch, which makes the effects on the gut microbiota intricate [46]. Type 4 resistant starch enriches *Bacteroides* and *Parabacteroides* spp. in the gut, while type 2 resistant starch increases *Ruminococcus bromii* and *Eubacterium rectale* spp. in humans and *Bifidobacterium*, *Akkermansia* and *Allobaculum* genera in mice [4,52,53]. Type 3 resistant starch is considered the most resistant form of resistant starch [54]. It has been shown to promote the growth of *R*. *bromii*, *E. rectale* and *Roseburia* spp. populations in the gut of different animal models and humans [16,55,56]. Moreover, *R. bromii* was suggested to be a keystone species in resistant starch degradation, particularly type 3, which is required for the other bacteria to utilize the products from resistant starch [57]. Concordantly to the other studies [16,55,56], the *Ruminococcus* genus (including *R. bromii*) increased in relative abundance in growing pigs that were fed with a type 3 resistant starch-containing diet [30]. An alteration in gut microbiome and a predominance of beneficial bacterial populations were observed in these pigs compared to control pigs. The enhanced beneficial bacterial populations included the metabolically reputable (e.g. SCFA-producing) populations of *Prevotella*, *Ruminococcus* and Lachnospiraceae, as well as others such as Veillonellaceae, *Bulleidia* and *Dialister* [30].

Another source of dietary fibre is algal polysaccharides such as alginates, agars and carrageenans from seaweeds, which are extensively used in the food industry as thickeners and stabilizing or emulsifying agents [58]. The most widely produced algal polysaccharide, alginate, is considered to be prebiotic [59,60]. This viscous dietary fibre confers many health benefits due to its gel-forming ability and other physicochemical properties, including fermentability by the gut microbiota [61]. These benefits include control of appetite, type 2 diabetes and obesity by enhancing satiety, refinement of gut barrier function and reduction of the damaging effects of luminal contents [35,58,61]. Alginate has been shown *in vitro* and i*n vivo* to be fermented at a low rate by the gut microbiota; however, its fermentability increases with time [58,60,62]. Moreover, it modifies the gut microbiota to a certain extent: an alginate-containing diet increased the relative abundance of some SCFA-producing populations such as *Roseburia*, *Ruminococcus* and *Lachnospira* in growing pigs, although the overall diversity and the composition of the gut microbiota remained unchanged [30]. Moreover, the number of bifidobacteria was increased by alginate in human subjects [63].

Fermentation of the complex carbohydrates by the gut bacteria results in beneficial effects for the host, which make them potential prebiotics. These benefits include a reduction in the formation of hazardous metabolites that are produced as a result of proteolytic activity [64]. Moreover, beneficial metabolites with anti-inflammatory and anti-cancer activities, such as phenolic compounds and SCFAs, are produced by gut bacteria from their fermentations [47,65].

## Bacteriocins

Antimicrobial substances produced by bacteria, i.e. bacteriocins, play a role in the competitive exclusion of pathogens, as well as interference competition among the gut microbiota, thereby helping the producers to colonize and establish a niche in the ecosystem [66,67]. In many cases, bacteriocins are better choices than traditional antibiotics because most bacteriocins have narrow-spectrum activity that can be used to remove unwanted bacteria and other pathogens without much disturbance to the commensal flora, in contrast to most antibiotics [68]. Furthermore, most antibiotics are enzyme inhibitors, inhibiting different biosynthetic pathways in cells, such as protein, DNA, RNA synthesis and cell-wall synthesis [69]. On the other hand, bacteriocins, especially those from Gram-positive bacteria, are membrane-active peptides, killing the target cell by membrane disruption [70]. Thus, antibiotic resistance mechanisms developed against antibiotics do not apply to them. Bacteriocins are therefore equally active against antibiotic-sensitive and antibiotic-resistant pathogens.

**Figure 1.**
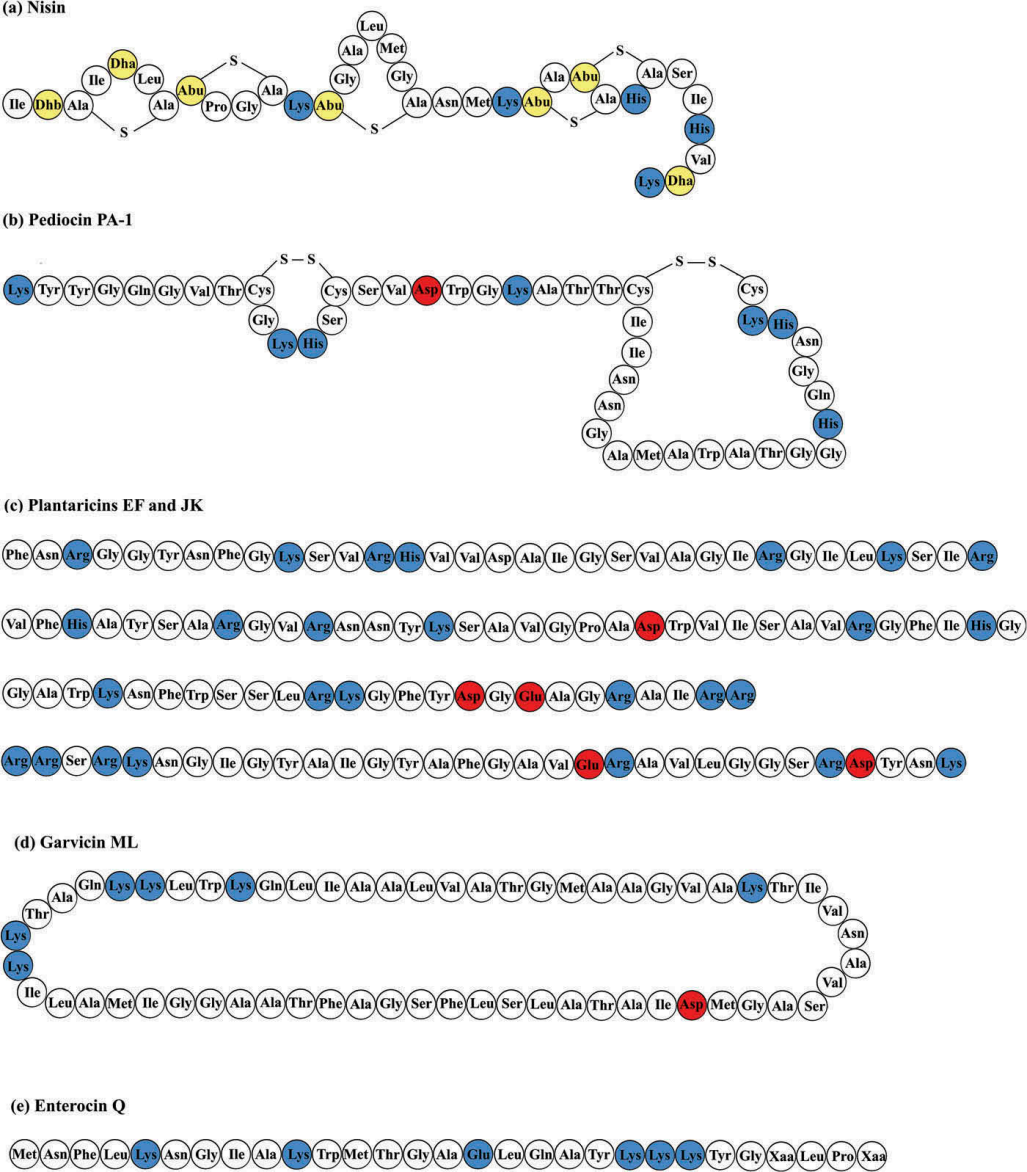
Amino acid sequences of representative bacteriocins from different classes: (a) nisin from class I [72]; (b) pediocin PA-1 from class IIa; (c) plantaricins EF and JK from class IIb [73]; (d) garvicin ML from class IIc [74]; and (e) enterocin Q from class IId [75]. The sequences of the peptides that form plantaricins EF and JK have been shown in the order of plantaricin E, plantaricin F, plantaricin J and plantaricin K. The red, blue and yellow amino acids represent acidic amino acids, basic amino acids and uncommon amino acids (Dhb, didehydroaminobutyric acid; Dha, didehydroalanine; Abu, 2-aminobutyric acid), respectively.

Bacteriocins are ‘bacterially produced, small, heat-stable peptides that are active against other bacteria and to which the producer has a specific immunity mechanism’ [71]. They are produced by a variety of microorganisms, i.e. Gram-positive and Gram-negative bacteria and some archaea [67]. The bacteriocins produced by Gram-positive bacteria, which are mostly lactic acid bacteria (LAB), are classified into two major groups: class I (lanthionine-containing bacteriocins/lantibiotics) and class II (non-lanthionine-containing bacteriocins) (Figure 1). Lantibiotics are post-translationally modified small peptides of 19–38 amino acids in length, which include the best known broad-antimicrobial spectrum bacteriocin, nisin [66,76]. Class II bacteriocins are non-modified or subjected to minor modifications, i.e. disulfide bond formation or circularization. This group of bacteriocins includes a heterogeneous class of small (30–70 amino acids), heat-stable peptides. Although the classification varies in the literature, they are divided into the following subclasses according to Cotter et al. [71]. Class IIa bacteriocins are known as pediocin-like bacteriocins, and have a relatively narrow antimicrobial spectrum. They are typically active against *Listeria*; nevertheless, their target cells also include *Enterococcus*, *Lactobacillus, Leuconostoc*, *Pediococcus* and *Clostridium* [77]. Class IIb bacteriocins are two-peptide bacteriocins that require the combined action of two different peptides with the encoding genes located next to each other in the same operon. These bacteriocins have often narrow-spectrum activity [73,78]. Class IIc bacteriocins are circular bacteriocins with the N- and C-termini covalently linked, which results in a cyclic structure [71]. Class IId is a miscellaneous group containing all other bacteriocins that do not fit into any of the aforementioned groups [71].

### Bacteriocins from LAB

Bacteriocins produced by LAB have attracted much interest in recent years because many bacteriocin producers in this bacterial group are probiotics. These microorganisms are commonly found in our food, especially in fermented products, and are therefore generally regarded as safe for human consumption [76]. Another common habitat of LAB is the gastrointestinal tract, where they develop complex means of molecular cross-talk with the host and other bacteria [79]. Bacteriocins are often seen as weapons, with a variety of inhibition spectra to compete with other bacteria that are likely to share the same niche. Most bacteriocins target species or genera closely related to the producers, while some can have much broader spectra [76,80]. Some LAB can compensate for the variety of the targets and the relatively narrow spectra of the bacteriocins by the production of multiple bacteriocins that belong to different classes [66].

### Impact of LAB bacteriocins on the gut microbiota

In the gut, bacteriocins may help the producer to survive and colonize, and inhibit the closely related competitive strains or pathogens, as well as influence the immune system of the host through their impact on gut microbial populations [80,81]. For instance, a *Lactobacillus salivarius* strain producing salivaricin P becomes predominant in porcine ileum when administered in combination with other probiotic strains that do not produce bacteriocins [82]. Antagonistic activity of the bacteriocin producers against a number of pathogenic or antibiotic-resistant bacterial strains in the gut has been a major focus of gut microbiota studies. Several LAB bacteriocins and/or bacteriocin-producing LAB have been shown to inhibit pathogens such as *Listeria monocytogenes* [83,84], *Clostridium difficile* [85–87], *Staphylococcus aureus* [88] and even *Salmonella enteritidis* [89], Some bacteriocin producers have also been reported to eliminate multidrug- or vancomycin-resistant enterococci [81,90]. Therefore, the production of bacteriocins may contribute to the beneficial activities in the gut.

Moreover, several studies have evaluated the effect of bacteriocin-producing LAB or their bacteriocins on the normal gut microbiota in live animals. For example, *L. salivarius* UCC118 producing bacteriocin Abp118 has been shown to cause significant but subtle changes in the murine and porcine intestinal microbiota [91]. A probiotic strain, *Lactobacillus plantarum* P-8, caused a shift in the faecal bacterial profile in humans, and this shift has been suggested to be due to the putative plantaricin production by this strain [92]. In another study, nisin F has been suggested to have a stabilizing effect on the bacterial populations in the gut of mice [93]. These studies illustrate the impacts of bacteriocins on the gut microbiota; however, they vary greatly in terms of the administration method, the model used in the experimental design and the use of proper negative controls. This makes it very difficult to attribute the observed changes to the bacteriocin or the bacteriocin producer, and to make an overall inference.

We have recently conducted a comprehensive study to assess the effects of five different bacteriocin-producing LAB strains (and their isogenic non-producing strains as negative controls) on the gut microbiota in healthy mice. The bacteriocins are produced by food- or gut-associated LAB and belong to different subclasses of class II bacteriocins: sakacin A (class IIa), pediocin PA-1 (class IIa), enterocins P, Q and L50 (class IIb and IId), plantaricins EF and JK (class IIb) and garvicin ML (class IIc) [94]. When analysing the microbial community in faeces, it was observed that the overall structure remained largely unaffected in different treatments. However, when looking at the lower taxonomic levels, some significant changes were observed with some bacterial phylotypes, especially in the treatments with bacteriocins that have relatively broad inhibitory spectra (e.g. enterocins Q and L50 and garvicin ML) (Table 1). Some of these changes can be regarded as beneficial; for example, some bacterial populations that include potentially problematic strains were inhibited, e.g. *Staphylococcus* by enterocins, Enterococcaceae by garvicin ML and *Clostridium* by plantaricins.

**Table 1.** Bacteriocin-associated and non-bacteriocin-associated modifications of class II bacteriocin producers.

	Sakacin A	Pediocin PA-1	Enterocins Q and L50	Plantaricins EF and JK	Garvicin ML
Bacteriocin-associated effect^a^	Leuconostocaceae **↑**	*Clostridium***↑**	Enterococcaceae **↑**	*Clostridium***↓**	Leuconostocaceae **↑**
			*Streptococcus***↓**		Lactococcus **↑**
			*Staphylococcus***↓**		Enterococcaceae **↓**
	Total LAB **↑**			Total LAB **↑**	Total LAB **↑**
Non-bacteriocin-associated effect^b^		*Pediococcus***↑**	*Lactobacillus***↓**		
		*Lactobacillus***↓**			
		*Streptococcus***↓**			
		Enterococcaceae **↓**			

^a^^ ^Observations in the presence of bacteriocin-producing strains only; ^b ^observations in the presence of both bacteriocin-producing and non-bacteriocin-producing strains.

LAB, lactic acid bacteria.

Adapted from [94].

This relatively broadened spectrum activity by the bacteriocins in the gut is surprising as these bacteriocins normally do not inhibit the indicated pathogens in laboratory conditions (i.e. in pure cultures). One possible explanation for this is that some other unknown factors, possibly, for example, defensins or reactive oxygen species from the host, or secondary metabolites (organic acids) from other bacteria, may have contributed with synergetic effects on bacteriocins. Clearly, further investigation is needed to provide conclusive answers.

Moreover, the proportion of LAB was increased in the presence of sakacin A-, plantaricin- and garvicin ML-producing bacteria. These traits of the LAB bacteriocins offer the opportunity for manipulation of the specific populations by bacteriocin producers at different levels and in different directions without disturbing the symbiotic inhabitants of the gut. The gut microbiota in mice differs from the one in humans in several ways, e.g. having higher numbers of *Lactobacillus*; however, this study has demonstrated the beneficial activities of the bacteriocin-producing LAB strains in a gut environment. Knowledge on the interactions between the bacteria in such an environment will lead to studies on the health applications of bacteriocins on humans.

Regarding antimicrobials, the inhibition spectrum (target specificity) is an important factor since antimicrobials with very broad spectra (such as antibiotics) may cause dysbiosis, perturbing the well-balanced gut microbiota [31]. Often the occurrence of dysbiosis depends on composition of the gut microbiota, the antimicrobial resistance genes within the gut community and the types of the antimicrobials applied during treatments [31,95]. The disturbed microbiota may lead to the overgrowth of pathogens, causing adverse effects on the host [31]. In this context, bacteriocins exhibit remarkable advantages over antibiotics owing to their relatively narrow spectra. They do not exert major disturbances on the commensal gut microbiota, which is important for normal gut functioning. For example, pediocin PA-1, which is very active against pathogenic *Listeria* spp., does not cause major changes to the gut microbiota in healthy mice or *in vitro* [94,96]. In addition, the great diversity of bacteriocins, in terms of their antimicrobial spectra and target specificity, can provide us with the opportunity to select certain bacteriocins with defined properties to deal with a specific pathogen or a group of pathogens. Bacteriocins are also superior to antibiotics when it comes to target specificity, non-toxicity to the host, antagonistic activity against important pathogens and the possibility of *in situ* production by probiotics [68].

### Administration of bacteriocins

Bacteriocins are of proteinaceous nature and are therefore easily degraded by proteases in the gastric juice. Consequently, the administration of bacteriocins into the gut environments is an important research field. The administration of bacteriocins via LAB producers, which endure well in acidic conditions in the stomach, enables us to circumvent proteolysis during gastric transit and to produce bacteriocins *in situ* in the large intestine. Nevertheless, *in situ* production of bacteriocins in the gut should be ensured because the complexity of the gut microbiota and its metabolic activities can greatly influence both the production and antimicrobial activity of bacteriocins, especially when bacteriocin production involves quorum-sensing-based regulation [97]. This is a regulatory mechanism used by bacteria to coordinate cell-density-dependent processes, often in response to changes in the environment [98]. Such a regulatory mechanism involves a secreted pheromone that serves as a signalling molecule to measure cell density. When the pheromone reaches a certain critical threshold concentration, i.e. a certain cell density, it triggers a phospho-relay reaction in cells that eventually results in an adaptive response, which may be the activation of a set of selected genes [99]. Quorum-sensing-based bacteriocin production is relatively common in Gram-positive bacteria including LAB, such as the production of nisin and plantaricins [97,98,100]. On the other hand, the administration of purified or synthesized bacteriocins via delivery systems such as encapsulated pills or particles has also been developed for food and medical applications [101].

## Personalized use of probiotics and prebiotics

Metabolic and functional phenotypes of the gut microbiota are dependent on the microbiota composition [102]. Individual differences in the microbiota composition can therefore have effects on how drugs [103] and food components [104] are metabolized, and consequently on how the health of the individual is affected.

Diseases and disorders, such as inflammatory bowel disease and irritable bowel syndrome, are associated with patients who have both a normal and dysbiotic gut microbiota [105]. Recent evidence suggests that the effect of dietary interventions on irritable bowel syndrome is dependent on the degree of dysbiosis [106]. It has also been shown that the glycaemic response in diabetic patients can be predicted based on the microbiota composition [107].

A major challenge in the application of interventions is the lack of consensus across different studies. A factor that may contribute to this lack of coherent results is differences in the composition and function of gut microbiota. This has been illustrated for probiotic application by our recent study showing that the effect of probiotics on prevention of atopic dermatitis is associated with the intrinsic microbiota in early infancy [108].

For future development of the aforementioned interventions, the personal gut microbiota must be taken into account. We therefore foresee benefits of more targeted and personalized approaches for future applications. This may lead to higher success rates in the substantiation of health claims related to the use of bacteriocins, probiotics and prebiotics.

## Concluding remarks

It is evident that the gut microbiota can be modulated to improve host health through some interventions. Prebiotics act as a substrate for a group of gut bacteria and lead to exploitative competition, while bacteriocins directly harm selected bacteria and lead to interference competition. Furthermore, both prebiotics and bacteriocins differ greatly in terms of their physicochemical properties and modes of action, and bacteriocins display different target specificities and width of inhibitory spectra. Thus, these substances can lead to different metabolic directions and different types of competition in the gut ecosystem. The resulting effects can be rather complex and should therefore be assessed empirically and carefully to obtain safe and beneficial outcomes. These interventions have great potential in therapeutic treatments, e.g. to modulate the microbiota from an unhealthy state to a healthy state by dietary fibres or by inhibiting unwanted bacterial phylotypes with certain bacteriocin producers. Moreover, modifications that enhance the growth of SCFA producers by prebiotics are often appreciated as these metabolites not only are an important energy source for the host but also appear to play a role in immune stimulation [3] and probably also in the signalling pathway between brain and gut (the brain–gut axis) [109].
